# Quantitative Evaluation of Fibrosis in IPF Patients: Meaning of Diffuse Pulmonary Ossification

**DOI:** 10.3390/diagnostics11010113

**Published:** 2021-01-12

**Authors:** Monica Palermo, Francesco Tiralongo, Giulio Distefano, Ada Vancheri, Mauro Giuffrè, Fabio Pino, Pietro Valerio Foti, Gianluca Sambataro, Carlo Vancheri, Stefano Palmucci, Antonio Basile

**Affiliations:** 1 Radiology Unit 1, Department of Medical Surgical Sciences and Advanced Technologies “GF Ingrassia”, University—Hospital Policlinico “G. Rodolico—S. Marco”, University of Catania, 95123 Catania, Italy; tiralongofrancesco91@hotmail.it (F.T.); giuliodistefano@gmail.com (G.D.); pietrofoti@hotmail.com (P.V.F.); spalmucci@sirm.org (S.P.); basile.antonello73@gmail.com (A.B.); 2 Regional Referral Centre for Rare Lung Diseases, Department of Clinical and Experimental Medicine, University—Hospital Policlinico “G. Rodolico—S. Marco”, University of Catania, 95123 Catania, Italy; adact1@hotmail.it (A.V.); fabius.90@hotmail.it (F.P.); dottorsambataro@gmail.com (G.S.); vanch-eri@unict.it (C.V.); 3 Department of Medical, Surgical and Health Sciences, University of Trieste, 34151 Trieste, Italy; gff.mauro@gmail.com

**Keywords:** lung diseases, interstitial lung diseases, IPF, fibrosis, HRCT, pulmonary ossification, visual score, quantification, histogram-based analysis, kurtosis

## Abstract

To investigate the role of diffuse pulmonary ossification (DPO) in disease severity in a population of Idiopathic Pulmonary Fibrosis (IPF) patients. This retrospective study was carried out on 95 IPF patients—44 with DPO on high resolution computed tomography (HRCT) and 51 with no calcifications detected on HRCT. Pulmonary Function Tests (PFTs) acquired nearest to the HRCT were collected. Images were analyzed by two radiologists using a qualitative method, based on HRCT fibrosis visual score, and using a quantitative method, based on histogram-based analysis. The Spearman’s rank correlation coefficient was used to measure the strength and direction of the linear relationship between HRCT fibrosis score and PFTs; in addition, Spearman’s rank correlation coefficient was used to explore the relationships between HRCT fibrosis score and quantitative index and between quantitative indexes and PFTs. A weak correlation between HRCT fibrosis score and PFTs was proven (r =–0.014 and *p* = 0.9347 for FVC (Forced Vital Capacity), r = −0.379 and *p* = 0.0174 for DLCO (Carbon monoxide diffusing capacity)). We found a moderate negative correlation between HRCT fibrosis score and kurtosis (r = −0.448, *p* = 0.004272) and skewness (r = −0.463, *p* = 0.003019) and a weak positive correlation with High Attenuation Area (HAA)% (r = 0.362, *p* = 0.0235). Moreover, a moderate linear correlation between Quantitative Indexes and FVC (r = 0.577, *p* = 0.000051 for kurtosis and FVC, r = 0.598, *p* = 0.000023 for skewness and FVC, r = −0.519, *p* = 0.0000364 for HAA% and FVC) and between quantitative indexes and DLCO (r = 0.469, *p* = 0.001508 for kurtosis, and DLCO, r = 0.474, *p* = 0.001309 for skewness and DLCO, r = −0.412, *p* = 0.005996 for HAA% and DLCO) was revealed. To better investigate the influence of DPO in disease progression, a longitudinal evaluation should be performed.

## 1. Introduction

As defined by the American Thoracic Society (ATS) and European Respiratory Society (ERS) in 2002, idiopathic interstitial pneumonias (IIPs) are a group of diffuse parenchymal lung diseases of unknown etiology, resulting from damage of the lung parenchyma by varying patterns of inflammation and fibrosis [1]. In 2013, the ATS and the ERS updated the 2002 classification, distinguishing major IIPs from rare IIPs and unclassifiable cases [2].

Major IIPs are grouped into “chronic fibrosing IP” (idiopathic pulmonary fibrosis—IPF and idiopathic nonspecific interstitial pneumonia—NSIP), “smoking related IP” (respiratory bronchiolitis-interstitial lung disease—RB-ILD and desquamative interstitial pneumonia—DIP), and “acute/subacute IP” (cryptogenic organizing pneumonia—COP and acute interstitial pneumonia—AIP). Rare IIPs include idiopathic lymphoid interstitial pneumonia (LIP) and idiopathic pleuro-parenchymal fibroelastosis (PPFE) [1].

IPF represents the most important diseases among IIPs; it has a poor prognosis and a poor survival (ranging from 2–3 years up to 5 years thanks to antifibrotic drugs) [3,4]. Its early diagnosis represents a diagnostic challenge; in addition, management and monitoring of IPF patients is necessary to evaluate disease’s behavior and proactively identify those with progressive course. As stated by the White Paper document, published by the Fleischner Society [5], chest high resolution computed tomography (HRCT) should be routinely performed at 1 year, in order to identify some complications of diseases.

**Figure 1:**
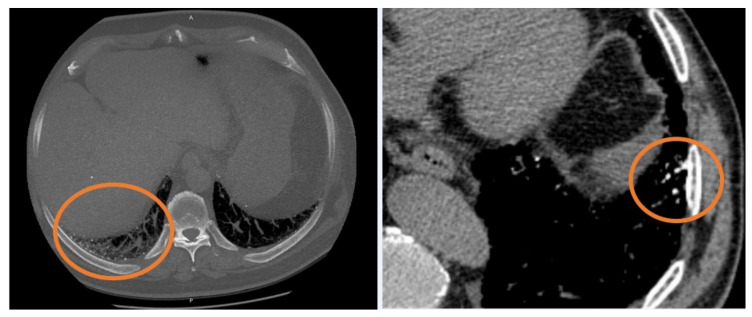
Two examples of dendriform ossification (orange circles), localized in subpleural regions.

Quantitative HRCT analysis has been proposed to characterize disease prognosis and stratify patients [6], while Oda, in 2014 [7], proposed the visual score, assessed on HRCT images using a semiquantitative analysis. This visual score may be considered a marker for monitoring disease evolution. In addition to this quantitative approach, several papers have recently investigated biological, clinical, and radiological markers that influence disease behavior [8,9,10]. In this regard, the influence of pulmonary ossification in disease evolution nowadays is still uncertain. Diffuse pulmonary ossification (DPO) is a rare form of ILD and it is less frequent in patients without a background of lung disease. DPO is characterized by small tiny calcifications, with or without bone marrow, located in the subpleural regions of lungs. Among the two forms (dendriform and nodular) described in literature, the dendriform DPO is more often associated with chronic obstructive lung disease and interstitial fibrosis (Figure 1) (Table 1) [11,12,13]. The presence of DPO can help the diagnose of Usual Interstitial Pneumonia (UIP) and it can be considered as an ancillary finding of fibrosis [14,15].

Pathogenic mechanisms of ectopic ossification, or rather, metaplastic mature bone in alveolar or interstitial spaces, enclose chronic inflammation, anoxia, angiogenesis, chronic venous congestion, and lung injury in fibrosis; an underlying parenchymal fibrosis is retained to be a potential “trigger” for DPO, causing all these changes in lung environment, creating chronic anoxia, which produces an acid environment, stimulating fibroblasts proliferation and metaplastic osseous formation [13,16,17]. In a recent study published by Egashira et al. [11], DPO was found to have a much higher prevalence in UIP patients than in other non-IPF fibrosing ILDs.

All of this considered, DPO could be evaluated as a marker of progressive fibrosis phenotypes.

Among the other causes of pulmonary ossification, the following should be mentioned: pulmonary amyloidosis, sarcoidosis, histoplasmosis, metastatic diseases (breast, osteogenic sarcoma, melanoma) and, as already outlined, preexisting cardiac disorders, such as mitral stenosis, which is a relatively frequent cause of nodular DPO [13].

Therefore, the aim of this study is to evaluate the influence of pulmonary ossification in IPF severity and evolution, carrying out a semiquantitative analysis (HRCT Visual Score) and a quantitative analysis (histogram based analysis) in two different sets of IPF patients, including subjects with DPO and subject without DPO.

## 2. Materials and Methods

### 2.1. Study Design, Setting, and Participants

This retrospective study was performed by the collaboration between the Radiology Institute of the University Hospital “G. Rodolico-San Marco” (University of Catania) and the Referral Centre for Rare Lung Diseases of University of Catania. All patients provided a personal consensus for collection and acquisition of data.

Inclusion criteria adopted in this study were:

(1) IPF diagnosis, according to the Fleischner Society guidelines published in 2018 [5]. IPF diagnosis was obtained after a multidisciplinary evaluation, involving radiologists, pneumologists, and rheumatologists;
(2) Diagnosis of IPF in the past 24–36 months and an IPF stage, based on GAP Index (Gender–Age–lung Physiology variables), of 1 or 2 [18];
(3) IPF patients, with at least one volumetric HRCT, showing tiny multiple branching calcifications, reproducing the so called “pulmonary ossification lung” or DPO, as defined by the definition 1 of Fleischner society Glossary: “10 or more bilateral nodular ossifications and/or small dense nodules” [19].

Patients were not included in our analysis in case of inadequate HRCT examinations, due to motion artefacts, axial acquisition, or in case of images, where acquired, with contrast medium administration, and in case of other causes of pulmonary calcifications.

According to the inclusion criteria mentioned, this study included 44 patients, also called cases—41 males (93.2%) and 3 females (6.8%), aged from 50 to 87 years, average age of 71); 88.6% of these patients were smokers or former smokers. All enrolled cases showed a UIP pattern (*n* = 29) or a possible UIP pattern (*n* = 15) on HRCT examinations.

Pulmonary Function Tests (PFTs), namely Forced Vital Capacity (FVC) and Carbon monoxide diffusing capacity (DLCO), were collected for each patient of our population case study; in more detail, we considered the PFT values nearest to the HRCT data acquisition (no more than 3 months).

Cases were compared to a control population composed by patients with IPF (according to points 1 and 2 of our inclusion criteria) and without the presence of ossification at HRCT; control patients were selected, according to a random criterion of enrolment, from our electronic Radiology Information System/Picture Archiving and Communication System (RIS/PACS). A total of 51 cases—40 males (78.4%) and 11 females (21.6%) were finally included for the statistical analysis, with an average age of 69, ranging from a minimum of 55 to a maximum of 85. Of these patients, 38 patients showed an UIP pattern, while 13 showed a possible UIP pattern on HRCT examination.

Demographic data and PTF values are summarized in Table 2.

### 2.2. Protocol Details

As previously reported, we included only patients with at least one volumetric HRCT; in more detail, technical parameters for imaging acquisition were: thin section CT images ranged between 0.62 and 1.5 mm; sharp kernel imaging reconstruction, contiguous or overlap images, or HRCT with spacing < 10 mm; no contrast media administration.

All images were transferred and/or uploaded in a dedicated workstation for visual scoring and reading (Advantage Workstation 4.6, provided by General Electric (GE) Healthcare, Milwaukee, WI, USA).

### 2.3. Image Analysis

#### 2.3.1. Visual Score

Digital imaging and communication in medicine (DICOM) images were anonymously analyzed by two radiologists with different experience in the field of chest imaging, respectively 4 and 18 years of expertise.

An “HRCT fibrosis score” was calculated, according to the paper reported in literature by Oda K. et al. in 2014 [7].

**Figure 2:**
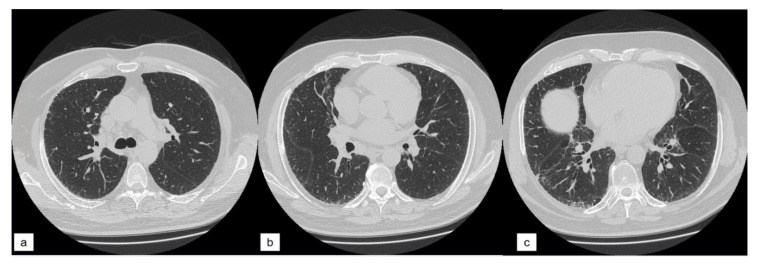
The three zones of high resolution computed tomography (HRCT) visual score. (**a**) “upper zone”, slice at the level of carina; (**b**) “middle zone”; (**c**) “lower zone” slice at the level of the inferior pulmonary vein.

The lung volume of each patient was divided into three different slices (Figure 2):

1. “Upper zone”: HRCT slice acquired at the level of carina;
2. “Middle zone”: HRCT slice acquired between the carina (“upper zone”) and the level of the inferior pulmonary vein (“lower zone”);
3. “Lower zone”: HRCT slice at the area of the lung below the level of the inferior pulmonary vein.

Lung pattern was classified into the four following categories and a grading scale score (1 to 4); each number represents the corresponding category number: (1) normal lung attenuation; (2) reticulations; (3) traction bronchiectasis; (4) honeycombing.

**Figure 3:**
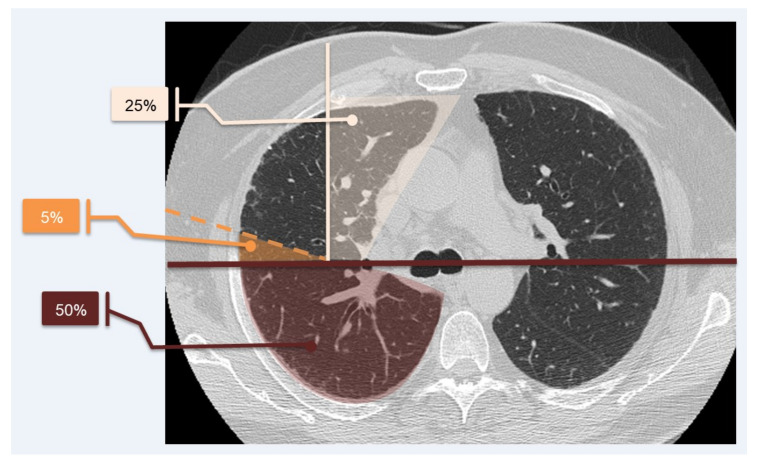
System of calculation of lung parenchyma percentage. Lung parenchyma of each level was divided by drawing a horizontal line (dark red line) and leaving a measurable area of 50%; a second line (white line), which ran perpendicularly to the horizontal one, left a 25% assessable area of each lung. Each 25% was subdivided into five portions, which corresponded to an area of 5% each (orange line divides 5% of parenchyma from the rest). This method helps with evaluating the percentage of the parenchyma with a specific pattern. The system was proposed by Sánchez et al. in 2018 [20].

To simplify calculations, we quantified the lung parenchyma in percentage, as proposed by Sánchez et al. in 2018 [20], as represented in Figure 3.

The score for each zone was calculated by multiplying the percentage of the involved area by the grading scale score (1–4). The six zone scores (three zones for the right lung and three for the left one) were averaged to determine the total score for each patient. Using this calculation method, the highest achievable score was of 400 points and the lowest score was of 100 points. The analysis was performed in two steps: in a first “reading analysis”, readers in a blinded mode reported their scores in a worksheet paper of excel; they were blinded for their respective reports. In the second step, they revised, in consensus, their scores. When reports were different among readers, a further evaluation was needed in order to reach, in consensus, a final score.

**Figure 4:**
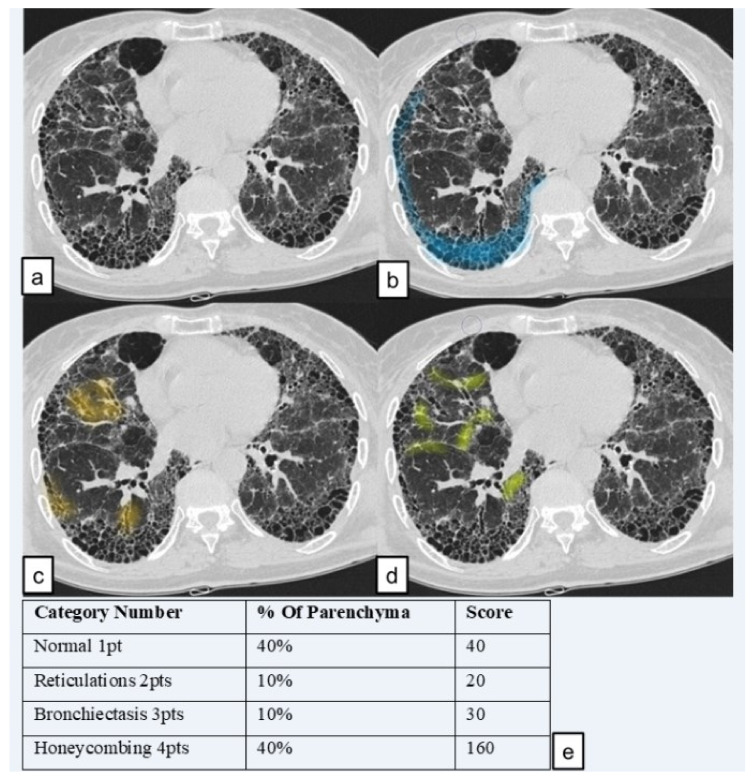
An example of HRCT score of “lower zone” of the right lung. (**a**) Slice of “lower zone” (**b**) in blue, area of honeycombing (**c**) in orange, area with traction bronchiectasis; (**d**) area of reticulation. (**e**) Scores of the slice (obtained by multiplying the category number with the affected percentage area).

We named the total score the “HRCT fibrosis score”. An example of how an HRCT fibrosis score was performed (in lower zone of right lung) is provided in Figure 4.

Therefore, six lung regions were finally analyzed by readers. For each examination, HRCT images were analyzed using the sharp kernel imaging reconstruction, using two windows pre-sets of visualization (lung parenchyma and bone window).

#### 2.3.2. Quantitative Analysis

**Figure 5:**
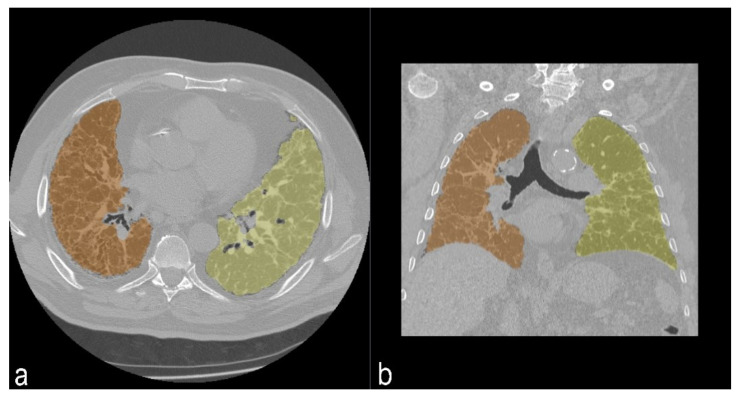
Segmentation of lung parenchima in axial view (**a**) and coronal view (**b**). In orange, segmentation of right lung; in yellow, segmentation of left lung.

The quantitative analysis was performed using 3D Slicer © (SlicerCIP4-8-1), an open source software platform for medical image informatics, image processing, and three-dimensional visualization. The lung volume was automatically segmented from the surrounding tissue and divided into three zones of the same size. This tool used an algorithm to isolate the lungs from other tissues and structures selecting pixels between −200 HU and −1.024 HU (Figure 5). Axial segmented images were then visually inspected by the readers and excluded if segmentation was inaccurate. Then, the HRCT attenuation histogram of the lungs was derived and specific parameters were automatically calculated. Quantitative CT indexes have been proposed as useful and standardized methods for analyzing and rating the extent of ILDs.

We chose to evaluate these specific following parameters, based on many recent works which analyzed quantitative indexes [21,22,23].

The parameters that were derived from histogram analysis and used were:

- Kurtosis;
- Skewness;
- High Attenuation Areas (HAA)%.

The kurtosis describes how sharply peaked a histogram is, when compared with the histogram of a normal distribution, which would have a kurtosis value of zero. It represents how tall and sharp the central peak is. A high value of kurtosis could indicate mild IPF, whereas a low kurtosis could indicate severe IPF. Skewness refers to the tendency of a distribution that determines its symmetry about the mean; it represents the asymmetry in a statistical distribution, in which the curve appears distorted or skewed either to the left or to the right. It represents the amount and direction of the skew. A perfectly symmetric distribution would have a skewness value of zero. A low skewness is correlated to a more fibrotic parenchyma and a more advanced disease. HAA% is indicative of parenchymal lesions, such as ground-glass opacity and reticulation. It was calculated as percentages of the extracted whole lung volume with attenuation values greater than 250 HU and less than 600 HU, as performed in the study by Ash et al. [6].

### 2.4. Statistical Analysis

According to the size of our sample, the Shapiro–Wilk test was performed to verify the normal distribution of variables. We explored inter-cohorts differences using the Mann–Whitney U test, given the non-parametric distribution of variables [24,25]. The Spearman’s rank correlation coefficient was used to measure the strength and direction of the linear relationship between HRCT fibrosis score and PFTs (FVC, DLCO); in addition, Spearman’s rank correlation coefficient was used to explore the relationships between HRCT fibrosis and quantitative indexes (kurtosis, skewness, and HAA%) and between quantitative indexes and PFTs. For all analyses, two-sided statistical significance was defined as *p* < 0.05. Data were analyzed using Statistical Package for Social Science (SPSS) version 25.0 (IBM SPSS Statistics for MAC OS. IBM Corp., Armonk, NY, USA).

## 3. Results

### 3.1. Correlation between HRCT Fibrosis Score and PFTs/Quantitative Indexes

The Spearman’s rank correlation coefficient revealed a weak linear correlation relationship between HRCT fibrosis score and FVC (r = −0.014; *p* = 0.9347); a weak linear negative correlation was reported between HRCT fibrosis score and DLCO (r= −0.379 (*p* = 0.0174).

Moderate negative correlations were found between HRCT Fibrosis Score and kurtosis (r = −0.448, *p* = 0.004272), and between HRCT fibrosis score and skewness (r = −0.463, *p* = 0.003019). A weak positive correlation was observed between HRCT fibrosis score and HAA% (r = 0.362, *p* = 0.0235).

### 3.2. Correlation between Quantitative Indexes and PFTs

**Figure 6:**
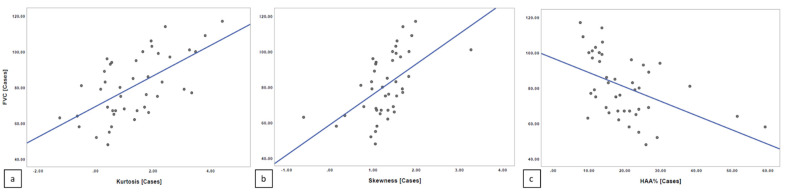
Spearman’s rank correlation among FVC and kurtosis (**a**), skewness (**b**), and High Attenuation Area (HAA)% (**c**) in diffuse pulmonary ossification (DPO) group.

The Spearman’s rank correlation coefficient revealed a moderate linear correlation relationship between quantitative index scores and PFT values, with r = 0.577 for kurtosis and FVC (*p* = 0.000051) and r = 0.598 for skewness and FVC (*p* = 0.000023); a moderate linear negative correlation was reported between HAA% and FVC, with r = −0.519 (*p* = 0.0000364) (Figure 6).

**Figure 7:**
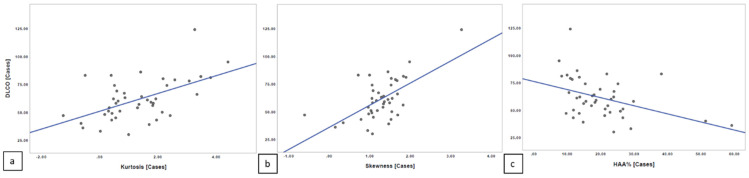
Spearman’s rank correlation among DLCO and kurtosis (**a**), skewness (**b**), and HAA% (**c**) in DPO group.

The Spearman’s rank correlation revealed lower values of correlation between quantitative indexes and DLCO. We found a moderate linear correlation relationship between quantitative index scores and PFT values, with r = 0.469 for kurtosis and DLCO (*p* = 0.001508) and r = 0.474 for skewness and DLCO (*p* = 0.001309). A moderate linear negative correlation was found between HAA% and DLCO, with r = −0.412 (*p* = 0.005996) (Figure 7).

### 3.3. PFTs and HRCT Fibrosis Score

For all of the patients included in this study, we evaluated respiratory functions with FVC and DLCO values achieved by spirometry test. Mean FVC value was 80.69 (±17.66) in the DPO group, and 90.92 (±18.87) in the group of patients with no DPO. Performing the Mann–Whitney U-test, there was a statistically significant difference between the two groups, with *p* = 0.005.

Mean DLCO value was 61.41 (±18.51) in the DPO group, and 63.58 (±16.44) in the control group; no statistical difference was observed comparing the two groups (*p* = 0.3).

The comparison with the Student’s t-test of HRCT fibrosis visual score between the two groups of patients showed no statistically significant difference, with a *p* = 0.357.

### 3.4. Histogram Based Analysis

The difference between the kurtosis of patients with DPO (mean = 1.3306, ± 1.264) and controls (mean = 3.2816, ± 2.26) was not statistically significant (*p* = 0.267). There were no statistically significant differences between skewness values in the two groups (*p* = 0.337), with mean value equal to 1.283 (± 0.568) in the DPO group, and equal to 1.326 (± 0.496) in the control group. There was no statistically significant difference for HAA% (*p* = 0.352) between cases (mean = 19.941, ± 10.4) and controls (mean = 18.642, ± 9.22), as reported in Table 3.

## 4. Discussion

IPF is a serious chronic disease with poor prognosis; despite new promising specific therapies, mortality rates remain high and functional disease progression is still worrying. As recently evaluated by Inoue et al., several biomarkers have the potential to become central to the clinical evaluation and monitoring of ILDs patients with a progressive phenotypes; among the analyzed biomarkers, levels of transforming growth factor-β levels (TGF-β), released by damaged epithelial cells, are increased in the lungs of patients with IPF, especially in UIP, and it could play an important role as a promoter of lung fibrosis and also as a stimulator for proliferation of chondrocytes and osteocytes [11,26,27,28].

In view of these considerations, identifying a morphological marker of disease, which can help in early IPF diagnosis and can be associated to progressive phenotypes, could make the difference for a more personalized management of the patient.

As mentioned before, many studies have investigated relationship between IPF and DPO, helping the differentiation of IPF from others interstitial lung diseases [17,29]; they have also revealed DPO highly associated to a higher grade of fibrosis and to poor survival in idiopathic NSIP. Thus, DPO could reflect the severity of the fibrosis [30].

Considering all these aspects, we expected that cases patients presented a higher grade of fibrosis. A moderate correlation between all quantitative indexes and PFTs in cases patients was proven (*p* < 0.05); kurtosis, and skewness were found to be lower in patients with DPO, and HAA% values were higher in DPO group compared to the highest value of no-DPO group, despite results were not statistically significant.

An explanation of no statistically significant results can be attributed to the small number of enrolled patients, related to rarity of DPO, which represents the major limitation of our study. To better investigate the influence of DPO in disease progression, a longitudinal evaluation should be performed. We plan to evaluate, in the near future, both baseline and follow-up CT scans of IPF patients with and without DPO, and of ones who showed DPO during follow-up, performing a visual and a quantitative assessment, to better understand the evolution of the disease.

HRCT visual score was found to have a weak correlation with HAA% and a moderate correlation with kurtosis and skewness in the cases group (*p* < 0.05), while its correlation with FVC was not statistically significant. HRCT fibrosis visual score showed to be a non-useful tool to assess the difference in terms of gravity of fibrosis between the two groups. It should be noted that the two groups were homogeneous, with UIP and possible UIP patients, so, in no extremely severe cases, patterns could be approximately similar in the analyzed slices; precisely, the HRCT fibrosis score assesses the percentage of a pattern in only six slices of the whole lung, while quantitative indexes study the whole parenchyma, so HRCT fibrosis score could lead to a less specific evaluation. Finally, HRCT fibrosis score is a visual assessment (“eyeballing”), limited by many factors, first of all operator experience and personal interpretation.

Our data cannot allow stating that DPO is a well-established marker of disease progression, but they confirm literature data, demonstrating that DPO helps to diagnose UIP, even in atypical presentation, and it can be considered a radiological ancillary sign of fibrosis.

## 5. Conclusions

We wanted to investigate if DPO could be considered a marker of disease severity in patients with IPF, since the presence of lung ossification could be caused from the presence of a more inflamed and anoxic environment. Focusing our attention on quantitative data, first of all, kurtosis seemed to be a worse condition (lower values of kurtosis) in patients with DPO compared to non-DPO patients; moreover, the DPO group showed generally worse values of HAA% and skewness, although the difference was not statistically significant. These findings are promising and deserve further evaluation and work, to replicate these associations in larger cohorts; moreover, it could be interesting to apply this evaluation in a prospective view, to determine important changes in follow-up HRCT and in outcomes in both groups. In conclusion, prognostic significance of DPO in IPF needs to be deeply evaluated.

## Figures and Tables

**Table 1:** Main differences between dendriform type and nodular type of pulmonary ossification (from Gielis J. et al., 2011 [12]).

	DENDRIFORM PO	NODULAR PO
**Radiographic findings**	Branching, dendritic, coral-like	Round with smooth contours, lobulated
**Histological reports**	In alveolar septa, tubular shape of bone	Within alveolar spaces, lobulated bone
**Marrow**	Present	Absent
**Clinical features**	Associated with chronic obstructive lung disease and interstitial fibrosis	History of passive congestion and cardiac problems

**Table 2:** Summary Table of demographic data and PFTs.

	CASES (*n* = 44)	CONTROLS (*n* = 51)	*p*-Values
Demographic data			
Age (years)	71.6 ± 6.6	69. ± 6.5	*p* = 0.92
Smoker (current vs. former)	*n* = 39 (88.6%) (31 vs. 8)	*n* = 45 (88.2%) (34 vs. 11)	*p* = 0.93
No smoker	*n* = 5 (11.4%)	*n* = 6 (11.8%)	
Treatment (pirfenidone)	*n* = 39 (88.6%)	*n* = 47 (92.1 %)	*p* = 0.25
Diagnosis			
UIP	*n* = 29 (65.9%)	*n* = 38 (74.5%)	*p* = 0.56
POSSIBLE UIP	*n* = 15 (34.1%)	*n* = 13 (25.5%)	
PFTs			
Forced Vital Capacity (FVC) (mean)	80.69 ± 17.66	90.92 ± 18.87	*p* = 0.66
Carbon Monoxide Diffusing Capacity (DLCO) (mean)	61.41 ± 18.51	63.58 ± 16.44	*p* = 0.42

**Table 3:** Histogram Based Analysis—values of the quantitative indexes between cases and controls.

	CASES	CONTROLS	*p*-Values
Kurtosis	1.330 ± 1.264	3.2816 ± 2.26	*p* = 0.267
Skewness	1.283 ± 0.568	1.326 ± 0.496	*p* = 0.337
HAA%	19.941 ± 10.4	18.642 ± 9.22	*p* = 0.352

## Data Availability

The data presented in this study are available on request from the corresponding author.
